# A Dynamic Breathing Lung Chip for Precise Evaluation of Inhaled Drug Efficacy and Airway Epithelial Responses

**DOI:** 10.1021/acsbiomaterials.4c01377

**Published:** 2024-12-01

**Authors:** Chao-Yu Liu, Ying-Ru Chen, Hsuan-Yu Mu, Jen-Huang Huang

**Affiliations:** Department of Chemical Engineering, National Tsing Hua University, Hsinchu 30013, Taiwan

**Keywords:** breathing lung, inhaled drug efficacy, airway-on-a-chip, respiratory, dynamic breathing

## Abstract

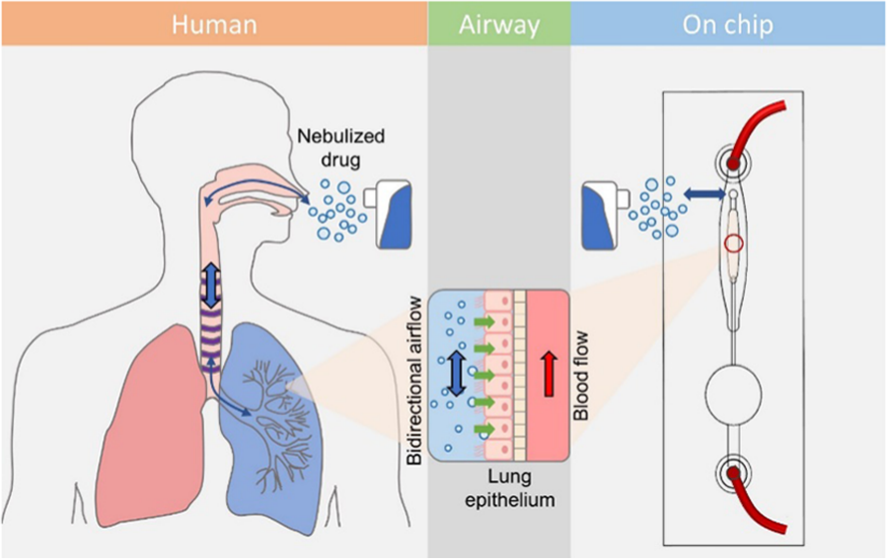

Inhaled therapy has become a crucial treatment option for respiratory diseases like asthma, cystic fibrosis, and chronic obstructive pulmonary disease (COPD), delivering drugs directly to bronchial and alveolar tissues. However, traditional static *in vitro* cell models, while valuable for studying pharmacokinetics (PK) and pharmacodynamics (PD), fall short in replicating the dynamic nature of physiological breathing. In this study, we present a breathing lung chip model that integrates a dynamic breathing mechanism with an air–liquid interface (ALI) culture environment to overcome these limitations. The platform replicates key aspects of lung physiology, including a functional airway interface, cyclic breathing motion, and medium circulation. Using the Calu-3 cell line to model airway epithelium, our experiments show that the incorporation of breathing motion significantly enhances the efficacy of inhaled drug delivery and cellular uptake, resulting in improved treatment outcomes compared to direct exposure of the drug. While further research is needed to explore its full potential, this platform holds promise for advancing inhaled drug screening and respiratory disease research.

## 1 Introduction

Breathing, a fundamental and essential physiological function, not only supports human life by providing oxygen and eliminating carbon dioxide but also holds significant potential for delivering pulmonary drugs. In recent years, inhaled therapy has emerged as a widely utilized approach for treating various respiratory diseases, including asthma, cystic fibrosis, and chronic obstructive pulmonary disease (COPD).^1,2^ By delivering drugs through the pulmonary route, localized treatment can be achieved in the bronchial or alveolar tissues without undergoing first-pass hepatic metabolism.^3^ The unique dendritic structure of the human lung, characterized by a large surface area and an extremely thin epithelium, facilitates the permeation and absorption of inhaled drugs into the circulation system. Moreover, the noninvasive nature of pulmonary drug administration and minimal side effects contribute to high patient compliance.^2,4^ However, the development of inhaled drugs faces several significant challenges, including a limited understanding of lung disease mechanisms, inadequate sustainability of drug release, and a lack of suitable drug screening models.^5^ Currently, traditional cell culture and animal models are commonly employed in preclinical tests to evaluate the pharmacokinetic (PK) and pharmacodynamic (PD) properties of potential drug candidates and predict their effects on the human body.^6^ While *in vivo* animal models are often considered the gold standard for assessing drug efficacy, they suffer from limitations such as high data variation and low similarity between species, thereby hindering their ability to accurately predict clinical outcomes.^7^ Moreover, the time-consuming and labor-intensive nature of *in vivo* models poses challenges for efficient drug development. Although conventional cell and tissue models serve as alternatives for evaluating drug transport, toxicity, and efficacy, the delivery of inhaled drugs in these models is typically limited to liquid formulations in culture media or simple deposition on the cell surface.^8^

To overcome these limitations, researchers have turned to lung-on-a-chip models developed using microfluidic techniques, which offer promising opportunities to simulate the physiological microenvironment and cellular architecture of the lungs. For example, Fishler et al. have emphasized replicating acinar particle dynamics and deposition, including models that incorporate true-scale acinar structures, breathing-like wall motion, and alveolar flow characteristics, providing more accurate predictions of inhaled particle fate and deposition patterns.^9^ These microfluidic lung-on-a-chip systems aim to bridge the gap between *in vivo* and *in vitro* models, leveraging their advantages of high physiological similarity and reproducibility to mimic lung physiology,^10,11^ disease conditions,^12^ and drug responses.^13^ However, most existing lung-on-a-chip models primarily focus on the functional alveolar-capillary interface, with limited attention given to airway-on-a-chip devices.^13,14^ Furthermore, the existing airway-on-a-chip models lack the critical breathing motion or are limited to one-way mechanical ventilation through the airway epithelium, making them less suitable for pulmonary drug screening.^10,15^ A notable exception is the work by Stucki et al., which developed a biomimetic diaphragm system for the alveolar space, enabling more accurate simulation of *in vivo* lung mechanics.^16^ Recent advancements, such as those by Sengupta et al., have utilized this approach for the nebulization of chemicals, further demonstrating the system’s potential in drug delivery studies.^17^ Further innovations in airway-on-chip platforms have enabled more accurate mimicking of physiological inhalation and particle deposition, helping researchers predict localized deposition outcomes for particulate matter and potential drug particles.^18^

In light of these challenges, we report a spontaneous breathing lung chip model incorporating the diseased human airway epithelium to investigate the cellular responses with and without the cyclic inhalation of nebulized drugs. Building upon our previous work on developing a hydraulic-driven breathing mechanism that mimics native human lung breathing focusing on aerosol deposition,^19^ this advanced model enables the cultivation of lung epithelial cells under dynamic air–liquid interface (ALI) conditions with adjustable airflow. Moreover, it also facilitates the visualization of inhalation drug deposition during the inhalation/exhalation process, enabling to obtain real-time images without sacrificing the chips. We show that the nebulized budesonide can be administered in aerosol form into the airway epithelium of the chip using the cyclic breathing mechanism, allowing for a comparative evaluation of the necessity of inhalation and exhalation mechanisms instead of direct exposure. Overall, the spontaneous breathing lung chip model is a promising tool to investigate the toxicity, efficacy, and delivery efficiency of inhalation drug candidates.

## 2 Materials and Methods

### 2.1 Fabrication of Breathing Lung Chip

The breathing lung chip was constructed from several components, including three 1.5 mm thick acrylic pieces, four 0.1 mm thick poly(ethylene terephthalate) (PET) sheets, an 85 μm thick flexible poly(dimethylsiloxane) (PDMS) membrane, a porous PET membrane (cut from SPLInsert Hanging, 37006, SPL, Korea) with 0.4 μm pore size, and two polycarbonate (PC) adaptors (BDMR210–9, Nordson MEDICAL) (Figure S1). The design of the device was initially generated using Solid Edge ST9 software (Siemens PLM Software) and subsequently converted for production through ULS Control Panel, which interfaced with a CO_2_ laser cutting machine (PLS4.75, Universal Laser System) for microchannel fabrication.^20^ Critical microfeatures of the device included:

(1) In the air layer, a channel designated for ALI cell culture measured 0.35 mm in height, 2 mm in width, and 11.5 mm in length. Additionally, a circular design with a 10 mm diameter facilitated the generation of cyclic airflow.
(2) In the medium layer, the primary channel was 1.7 mm deep, 4 mm wide, and 28.5 mm long, serving the purpose of medium storage and flow. A restricted channel, 10 mm in length and 0.5 mm in width, was designed to create the necessary pressure differential by regulating the fluid flow resistance.

To ensure the secure assembly of the plastic sheets and membranes, each acrylic and PET layer was pretreated with adhesive tape (9122, 3M, USA) before laser machining. Subsequently, these layers were carefully aligned and bonded using a rolling tool and alignment gigs, ensuring leak-free integrity throughout the experimental procedures. Finally, the assembly was completed by attaching the PC adaptors using epoxy glue.

### 2.2 Setup of Breathing Lung Chip

The breathing lung chip was configured by connecting the medium inlet and outlet of the chip to a peristaltic pump (pump head: RUNZE Fluid, China; motor: DMX-J-SA-17, Arcus Technology) using silicone tubing (1/16″ O.D., ULTRA-C-062–1F, Saint-Gobain, France). The peristaltic pump was controlled through computer-based software (Arcus Technology). It operated in cycles of 2 s pumping followed by 2 s rest, creating a cyclic breathing mechanism.

### 2.3 Characterization of Breathing Lung Chip

A differential pressure system was employed to analyze the airflow pattern to monitor pressure changes within the chip (Figure S2A). Initially, the breathing lung chip was connected to a 3-way valve with male and female integral lock rings (MTLL004–6005, FTLL004–6005, Nordson MEDICAL). This valve was further connected to a differential pressure transmitter 984 (Beck, Germany), while the third port was utilized for pressure balance. Data acquisition was achieved through a portable data acquisition module (USB-4718, Advantech, Taiwan) and transferred to specialized software (DAQNavi Data Logger, Advantech, Taiwan) for real-time monitoring.

For characterizing the airflow rate within the breathing lung chip, a 0.5 cm oil plug was introduced into the silicone tubing, which was then connected to the chip’s airflow port (Figure S2B). During the cyclic breathing process, the movement of the oil plug was recorded using video (Video S1) and subsequently analyzed to determine the airflow rate (*Q*_air_). This rate was calculated using eq 1.
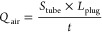
1where *S*_tube_ represents the cross-sectional area of the tubing, *L*_plug_ signifies the distance traveled by the oil plug, and *t* indicates the travel time of the oil plug.

### 2.4 Cell Culture in Breathing Lung Chip

Human airway epithelial cells (Calu-3) were generously obtained from Prof. Cheng-Wen Lin’s Lab (China Medical University, Taiwan) and maintained in Eagle’s minimum essential medium (EMEM, ATCC 30–2003TM) supplemented with 10% fetal bovine serum (F7524, Sigma), and 1% penicillin-streptomycin (SV30010, Cytiva). The Calu-3 cells were obtained from passage 26 and discarded after passage 40. The cells were cultured in a T75 flask and kept in an incubator at 37 °C with a 95% humidified atmosphere and 5% CO_2_ for later use. Before seeding the cells, all channels in the breathing lung chip were rinsed using PBS and sterilized by CoolCLAVE Plus Ozone and UV Sterilizer (E330110, Genlantis). To improve the adhesion of the cell to the porous membrane, the rat-tail type I collagen (A1048301, Thermo Fisher Scientific) was used before the cell seeding. Collagen was diluted to 50 μg/mL in 20 mM acetic acid with the amount of 5 μg/cm^2^ and then introduced onto the porous membrane through the seeding hole, followed by incubating at 37 °C for 1 h. After the incubation, the membrane was carefully rinsed 3 times with sterile PBS to remove the extra collagen. The collagen-coated device was used immediately or stored at 4 °C for future use.

### 2.5 Airway Epithelial Cell Culture

To establish the airway epithelium within the chip, a suspension of Calu-3 cells (20 μL, density: 8100 cells/μL) was introduced onto the collagen-coated membrane through the designated cell seeding hole. The effective culture area of the breathing chip measures 0.2026 cm^2^ (Figure S3A). For comparison, Calu-3 cells were also cultured in 12-well transwell inserts with a culture area of 0.33 cm^2^, maintaining the same seeding density of 8 × 10^5^ cells/cm^2^. The device and the transwell were cultured in an incubator at 37 °C with 5% CO_2_ for 24 h. Following this incubation period, the medium on the apical side was carefully aspirated to initiate the ALI culture (Figure S3B). The cell seeding hole was sealed using the Scotch tape (3M, USA), leaving an opening for airflow (Figure S3C). During ALI culture, the chip was placed in a Petri dish containing 2 mL of sterile PBS to maintain the moisture in the apical surface of the epithelium. Calu-3 medium in the medium layer was replenished every day. For airway inflammation modeling, TNF-α (300–01A, Peprotech, Israel) was added in the medium layer after 5 days of ALI culture to induce the inflammatory responses of Calu-3 and analyzed after 24 h of stimulation.

### 2.6 Cell Viability and Proliferation Assays

Live/Dead cell viability assay (CBA415, Sigma-Aldrich) was used to distinguish live cells from dead cells by offering different color fluorescence. The staining reagent was prepared by adding 2 μL of calcein-AM (Solution A) and 1 μL of propidium iodide (Solution B) in 1 mL of PBS. The cultured cells were washed twice with PBS, and 1 mL of staining reagent was added to the breathing lung chip. The chip was incubated at 37 °C for 15 min and observed under a fluorescence microscope (iRiSTM Digital Cell Imaging System, Logos Biosystems, Korea). For cell proliferation assay, Calu-3 cells were first harvested by using 20 μL of Trypsin 0.25% solution (SH4003.1, Cytiva) from three individual chips and transferred in a 96-well cell culture plate (TPP, Switzerland) with an 80 μL culture medium. The 10 μL of Cell Counting Kit-8 (CCK-8) reagent (Cyrusbioscience, Taiwan) was added to each well, and the 96-well plate was incubated at 37 °C with 5% CO_2_ for 1 h. The OD value for each well was obtained using a microplate reader (SpectraMax M2, Molecular device) at wavelength 450 nm, and the cell viability was calculated based on the OD measurement.

### 2.7 Immunostaining

Cells were fixed with 4% paraformaldehyde in PBS for 10 min at room temperature and washed with PBS 3 times. Cells were permeabilized with 0.02% (v/v) Triton X-100 for 10 min, followed by a blocking solution of 1% bovine albumin serum (BSA) in PBST (PBS + 0.1% ZO-1 (dilution = 1:25, 610966, BD Biosciences)) antibodies at 4 °C overnight. Goat antimouse AF488 (dilution = 1:200, ab150113, Abcam, U.K.) secondary antibodies were used for 1 h at room temperature in the dark. After washing, cells were incubated with 0.1 μg/mL DAPI for 3 min. Images were taken with a fluorescence microscope (iRiSTM Digital Cell Imaging System, Logos Biosystems, Korea).

### 2.8 FITC-Dextran Permeability Assay

To quantify the permeability of the airway epithelium, 1 mg/mL of FITC-dextran (4 kDa, 46,944, Sigma-Aldrich) was prepared in a cell culture medium. First, the membranes with or without cells were washed with the culture medium. Subsequently, 300 μL of culture medium was added to the medium layer of the chip, and 20 μL of FITC-dextran solution was added to the air layer, followed by incubation at 37 °C for 1 h. The medium in the medium layer was transferred to black 96-well microplates for analysis. The apparent permeability coefficient (*P*_app_) in cm/s of FITC-dextran diffusion across the epithelium was calculated using eq 2.^21^
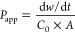
2where *w* is the accumulated FITC-dextran in the medium layer in mg, *t* is the duration of the permeability assay in second, *C*_0_ is the initial concentration of FITC-dextran in the air layer in mg/cm^3^, and *A* is the culture area of the breathing lung chip (*i.e.*, 0.2026 cm^2^).

### 2.9 Enzyme-Linked Immunosorbent Assay (ELISA)

MUC5AC was collected from the apical surface of airway epithelium using cold PBS washing several times. 120 μL for each sample were stored at −20 °C before analysis. IL-8 is the pro-inflammatory cytokines that can be produced and released by inflammation cells. Both were collected from the medium layer. Samples were also stored at −20 °C before analysis. The human MUC5AC and IL-8 levels were measured using ELISA kits (ABclonal) according to the manufacturer’s protocol.

### 2.10 Pulmonary Drug Delivery and Evaluation

The pulmonary drug was introduced into the transwell or chip using the vibrating mesh nebulizer (MBPN002, Pocket Air, Recare Medical, Taiwan) with a mass median aerodynamic diameter (MMAD) of about 4.2 μm to investigate the drug efficacy. The nebulizer was connected to a customized inhalation exposure chamber to ensure a well-distributed nebulization environment similar to the actual inhalation treatment (Figure S4). The chamber has a silicone gasket and a porous filter (0.22 μm in pore size, cut from T-25 flask, 90076, TPP, Switzerland) to create an isolated and air pressure balance environment for inhalation drug test. The transwell or breathing lung chip was placed in the chamber before treating the pulmonary drug. The treatment procedure was initiated once the nebulizer was turned on.

To calculate the dosage of the nebulized drug deposited in the channel of the chip, the fluorescein sodium solution (1.25 mg/mL, Sigma-Aldrich) was added in the medicine cup of the nebulizer for tracking of aerosol distribution in the breathing lung chip. For the drug administration experiment, 30 μM of budesonide solution was prepared in PBS with 0.1% DMSO and delivered using the nebulizer. All analyses were conducted on day 6 of ALI culture.

### 2.11 Statistical Analysis

The statistical analysis used GraphPad Prism (GraphPad Software Inc.) with unpaired Student’s *t* test. All results are presented as mean ± standard deviation of at least three independent determinants. Group differences were considered statistically significant when *P < 0.05, **P < 0.01, ***P < 0.001.

## 3 Results and Discussion

### 3.1 Principle and Design

**Figure 1:**
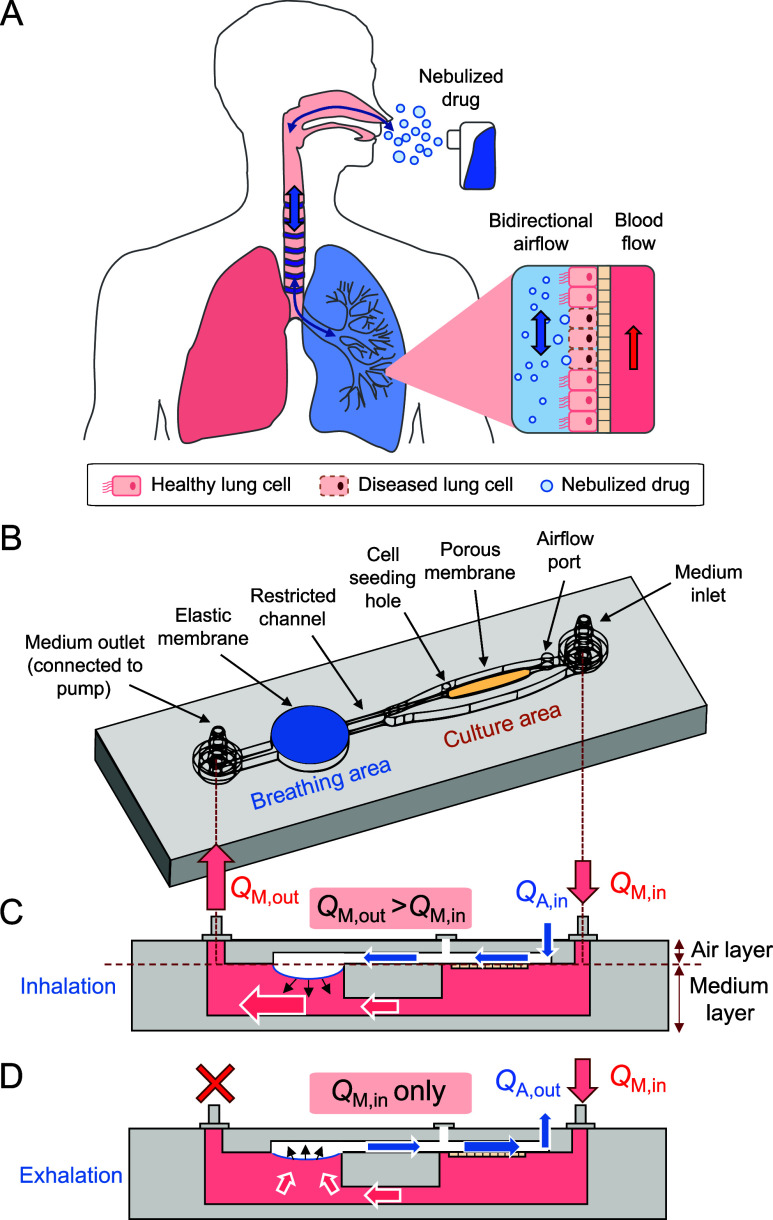
Working principle and design of the breathing lung chip. (A) Schematic of the nebulized drug delivery in the lung. The drug can flow in and out of the lung *via* bidirectional airflow and deposit on the lung epithelium. The drug molecule can deposit on the lung epithelium and interact with the diseased cells. (B) The design of the breathing lung chip. The chip contains breathing and culture areas, which can provide the cyclic breathing motion and culture of the lung cells. (C) The inhalation process of the breathing lung chip. The inflation of elastic membrane caused by the medium allows the air to flow in, enabling the air to flow in. (D) The exhalation process of the breathing lung chip. The deflation of the elastic membrane due to the contraction allows the air to flow in, enabling the air to flow out.

Nebulized drug delivery stands as a highly targeted method for administering drugs directly into the respiratory system through the utilization of a nebulizer device. By converting liquid drugs into an aerosol form, nebulization ensures the drugs can effectively reach the lungs for treatment (Figure 1A). This transformation is achieved through compressed air or ultrasonic vibrations, resulting in the medication as a fine mist or aerosol. Subsequently, patients inhale this aerosolized medication, allowing it to deposit onto the surfaces of the respiratory tract. Following deposition, the drug can be readily absorbed by the respiratory epithelium and ultimately enter the bloodstream, providing local or systemic therapeutic effects. To comprehensively investigate the toxicity, efficacy, and delivery efficiency of nebulized drugs, the breathing lung chip must encompass the vital features of cell culture and cyclic breathing. This combined feature enables the cells to be exposed to a bidirectional airflow, representing the back-and-forth movement of air that occurs during inhalation and exhalation within the airway (Figure 1B). Cyclic breathing is a fundamental characteristic of lung physiology and is pivotal in effectively distributing pulmonary drugs.^22^ To replicate this biomimetic breathing motion, the design of the breathing lung chip was inspired by the physiological breathing of human beings. During the inhalation phase, the contraction of muscles and diaphragm expands the chest, creating a partial vacuum that draws air into the lungs. Conversely, during exhalation, the relaxation of muscles and the diaphragm reduces chest volume, allowing air to be compressed and expelled from the lungs.

To harmonize cell culture and cyclic breathing features within a single chip, the breathing lung chip device was ingeniously designed with dedicated breathing and culture areas (Figure 1C). The elastic membrane within the breathing area serves as the mechanism for the inflow and outflow of air, while the porous membrane within the culture area permits the culture medium to permeate from the medium layer into the air layer of the chip, thus creating an ALI culture environment for bronchiole cells (Figure 1D). During the inspiratory phase, the withdrawal of the culture medium from the chip results in the outlet flow rate (*Q*_M,out_) exceeding the inlet flow rate (*Q*_M,in_) due to a restricted channel in the medium layer. This differential pressure within the medium layer causes the elastic membrane to deform, generating a temporary vacuum and facilitating spontaneous airflow (*Q*_A,in_) into the chip. Conversely, during the expiratory phase, the withdrawal of the culture medium ceases, and only the medium from the inlet enters the chip to balance the pressure differential. This allows the elastic membrane to return to its original state and prompts the outflow of air (*Q*_A,out_) from the chip. Consequently, the breathing process can be meticulously controlled by manipulating the operating time of the pump. In this study, the breathing cycle was set at 15 breaths per minute, with the pump operating for 2 s and then resting for 2 s. This design aligns with the typical adult respiration rate, which falls between 12 and 20 breaths per minute. Importantly, it provides a remarkable replication of the back-and-forth airflow observed during human respiration, thereby creating an environment conducive to entering nebulized drugs into the airway under physiological conditions.

### 3.2 Cyclic Breathing and ALI Culture in Breathing Lung Chip

**Figure 2:**
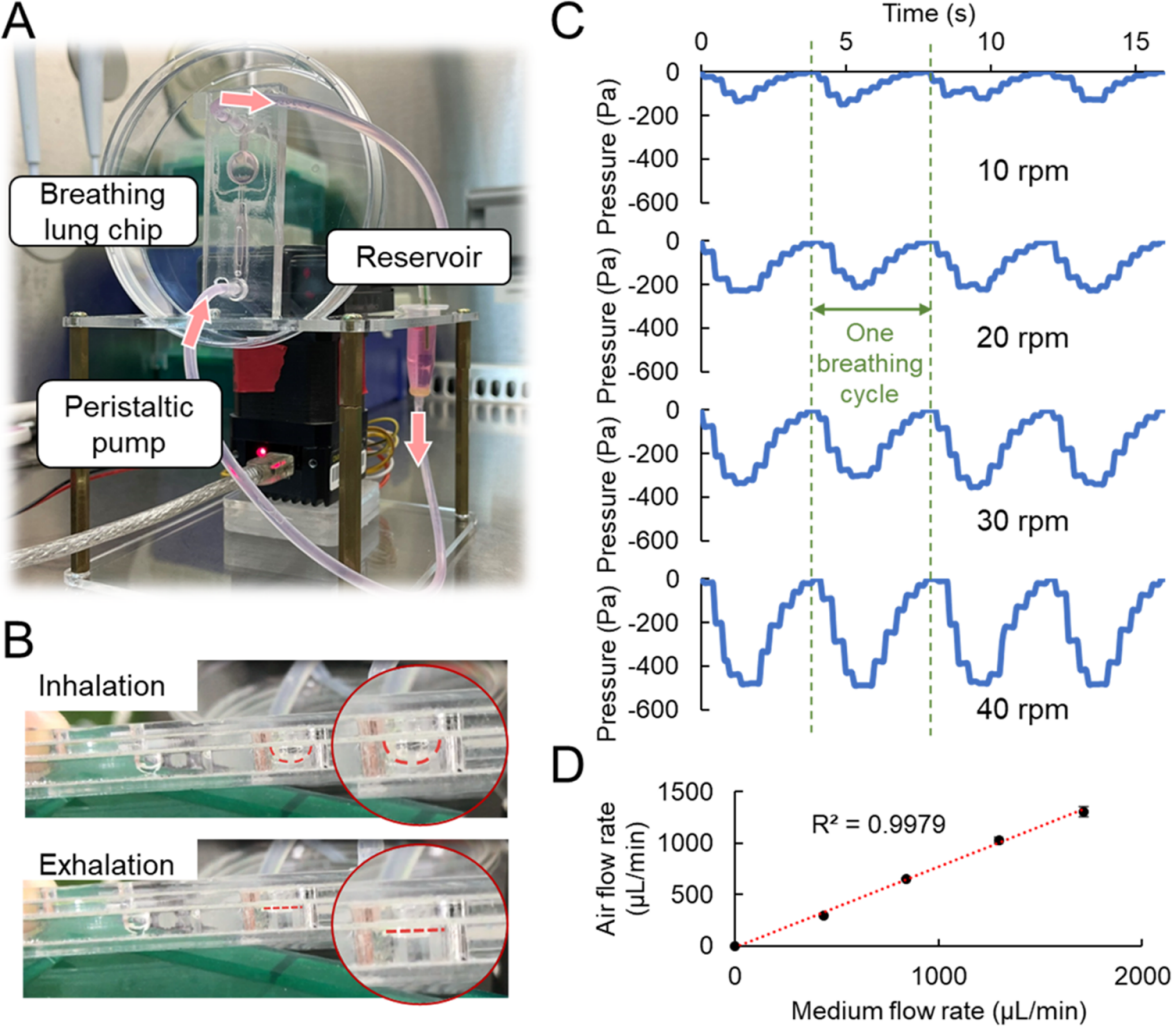
Characterization of breathing lung chip. (A) Setup of breathing lung chip system. The system comprises a breathing lung chip, a peristaltic pump, and a medium reservoir. (B) Side view of inflation and deflation of the elastic membrane in the chip during the inhalation and exhalation processes. (C) The cyclic pressure-time profiles under different rotation speeds. One breathing cycle is 4 s (2 s for inhalation and 2 s for exhalation). (D) The relationship between medium flow rate and airflow rate (*n* = 3).

To enable simultaneous cell culture and cyclic breathing, the breathing lung chip was integrated with a peristaltic pump and a medium reservoir, establishing a circulating medium flow system (Figure 2A and Video S2). This configuration allowed the cells to be cultured under dynamic ALI conditions. Figure 2B shows that the pressure differential inside the chip drove the inflation and deflation of the PDMS membrane, facilitating air inhalation during inflation and exhalation during deflation (Video S3). To modulate these processes, the rotational speed of the peristaltic pump could be adjusted with the aid of a computer program, thereby creating varying degrees of pressure differential within the chip (Figure 2C). Each cycle of negative pressure, spanning 4 s, encapsulates one complete breathing cycle, comprising 2 s inhalation followed by 2 s exhalation. Notably, the pressure within the chip was amenable to manipulation by controlling the rotation speed of the pump.

Subsequently, the airflow rate was evaluated and exhibited a proportional relationship with the medium flow rate (or pump rotation speed). This observation indicates that the breathing pattern, magnitude, and frequency can be precisely controlled to meet the specific requirements for inhalation drug applications (Figure 2D). Furthermore, the hydraulic diameter (*D*_h_) of the airway within the air layer of the chip was deliberately designed to measure 0.49 mm. This dimension aligns with the 16th generation of Weibel’s lung model and carries significance due to its strategic location just before the entry of nebulized drugs into the respiratory (alveolar) zone.^23^ The wall shear stress, a critical parameter, was calculated using the Poiseuille model, with the specific formula provided in eq 3.^24^
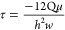
3where τ is the shear stress, *Q* is the volumetric flow rate, μ is fluid viscosity, *h* and *w* represent channel height and width, respectively. These meticulous design and measurement considerations ensure that the breathing lung chip possesses the necessary capabilities to simulate physiological breathing, thus offering an ideal platform for investigating inhalation drug applications.

Consequently, we calculated the Reynolds number (*Re*) and shear stress (τ) of the airflow at varying rotation speeds (Table 1). We aimed to generate an airflow (724 μL/min) that accurately mimics the conditions of the 16th generation of the airway (*Re* = 0.64 or air speed = 2.23 cm/s) for a 75-kg human. This representation was pivotal for the upcoming experimental phases. We selected a peristaltic pump rotation speed of 22 rpm (equating to a flow rate of 928 μL/min) to achieve this. A laminar airflow was consistently generated within the microchannel at this specific medium flow rate, characterized by a predictable parabolic velocity profile. This choice ensured the chip’s airflow matched the human airway’s physiological characteristics. To guarantee the reliability and stability of the breathing lung chip, assessments were conducted to ascertain that the chip maintained a consistent breathing condition for drug testing. As illustrated in Figure S2, a uniform cyclic breathing motion pattern was observed in three independent breathing lung chips. This consistency affirmed that a sustained and consistent breathing motion could be maintained at the same medium flow rate for at least 5 days, thus confirming the suitability of the chip for long-term usage.

**Table 1:** *Re* and Shear Stress in the Airway of the Chip at Different Pump Speeds

pump speed (rpm)	*Q*_M_ (μL/min)^a^	*Q*_A_ (μL/min)^b^	*Re*	τ (dyn/cm^2^)
10	435	297	0.269	0.045
20	840	653	0.593	0.099
30	1296	1030	0.934	0.156
40	1712	1307	1.186	0.198

a The medium flow rate was obtained according to calibration curve in Figure S5.

b The airflow rate was obtained using eq 1.

### 3.3 Formation of Airway Epithelium

In respiratory research and pharmaceutical development, the accurate replication of airway epithelium on lung-on-a-chip devices has emerged as an auspicious approach. We selected the Calu-3 cell line as the primary candidate for airway epithelium cultivation due to its notable expression of critical tight junction proteins, particularly ZO-1 and E-cadherin. While it is well recognized that Calu-3 cells possess phenotypical limitations compared to primary bronchial epithelial cells, they still exhibit several characteristics that make them suitable for studying tight junction formation and cell barrier functions. Additionally, Calu-3 cells exhibit essential features such as mucus production, cellular differentiation, and the expression of transport proteins, providing a comprehensive and dependable representation of the complex airway epithelium.^25,26^ Furthermore, the extensive utilization of Calu-3 cells in assessing drug efficacy underscores their established significance in pharmaceutical research.^27^

**Figure 3:**
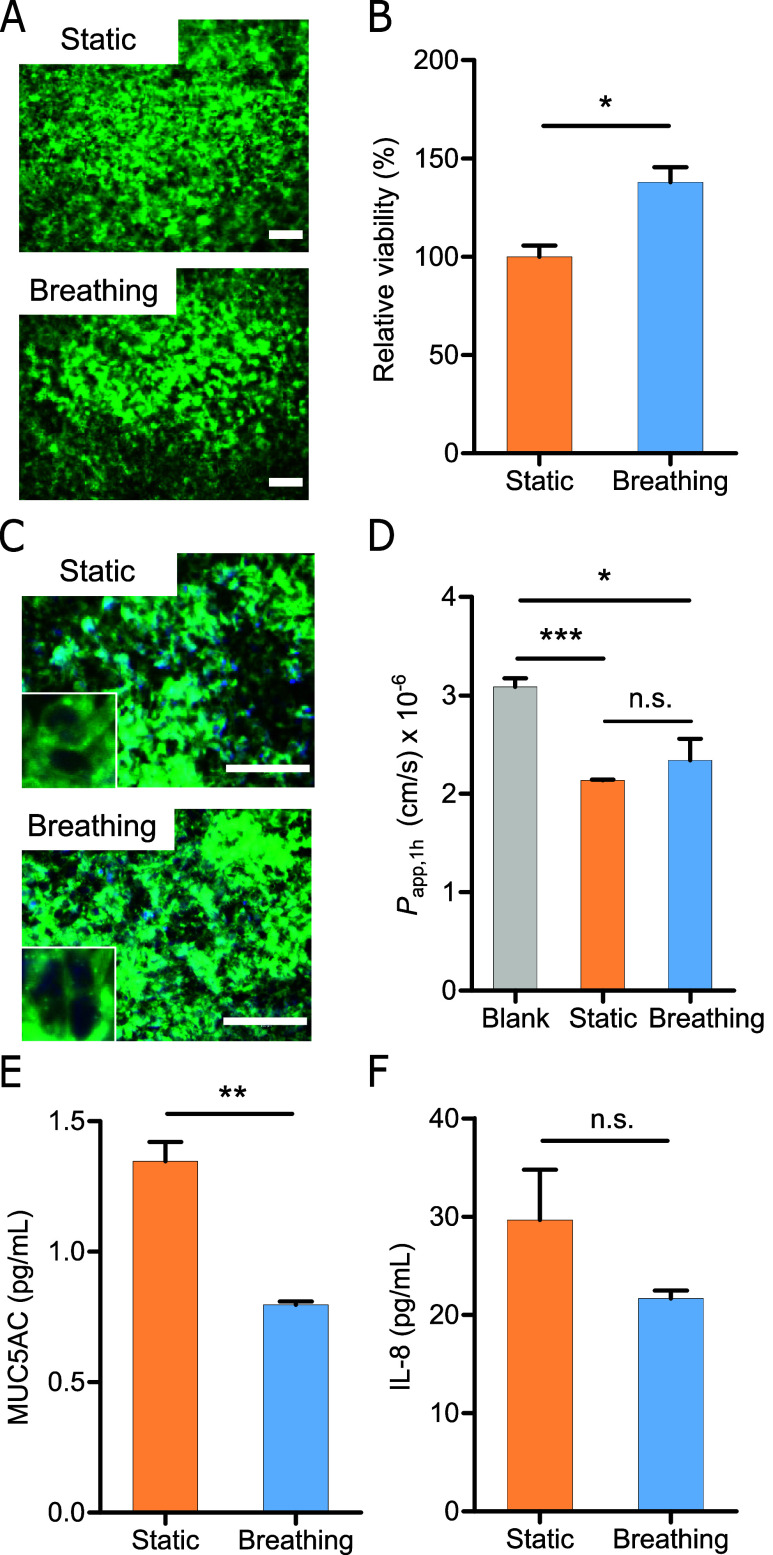
Cell culture in breathing lung chip. (A) Live/dead staining of the cells cultured at static condition (without breathing) and breathing culture condition for 4 days of ALI culture. Green: live cells; red: dead cells. Scale bar = 200 μm. (B) Cell viability of Calu-3 cultured at static and breathing conditions (*n* = 3). (C) Immunofluorescence staining images of ZO-1 (green) in DAPI (blue)-stained Calu-3 at static and breathing culture conditions. Scale bar = 100 μm. (D) Permeability of FITC-dextran in the blank (without cells), static, and breathing culture conditions of the breathing lung chip (*n* = 3). (E) MUC5AC secretion of Calu-3 cultured at static and breathing conditions. (F) IL-8 release of Calu-3 cultured at static and breathing conditions (*n* = 3). *p < 0.05, **p < 0.001, ***p < 0.0001. All experiments were conducted using an airflow rate of 724 μL/min, corresponding to an air speed of 2.23 cm/s for breathing culture conditions.

After seeding the Calu-3 cells within the breathing lung chip, the cells were cultured at a liquid–liquid interface (LLI) for 1 day to facilitate proliferation. Subsequently, the medium in the air layer of the chip was removed to establish an ALI culture environment. The cells were then cultured for an additional 4 days, with or without the inclusion of breathing motion. A cell viability analysis was conducted to confirm the ability of the cells to proliferate and maintain viability in both static and breathing motion cultures (Figures 3A and S6). The results of a CCK-8 assay confirmed that Calu-3 cells cultured under breathing conditions exhibited higher viability compared to those in static culture, suggesting that airflow may promote cell growth (Figure 3B). However, we also acknowledge that the observed increase in viability could be attributed to higher metabolic activity as a rescue response to breathing-induced stress, rather than solely to enhanced cell growth. Further investigations will be needed to differentiate between these potential mechanisms. In conditions of relatively high humidity and comfort, cells may not need to produce mucins, as cyclic breathing conditions more closely resemble physiological conditions than static cell cultures.

Immunostaining of the ZO-1 protein revealed that ALI cultures under static and breathing conditions could form tight junctions (Figure 3C). The epithelial integrity was further assessed by measuring the apparent permeability coefficient, *P*_app,1h_, using FITC-dextran as a permeation marker after 1 h treatment. The results demonstrated that cells cultured under both static and breathing conditions effectively prevented the penetration of FITC-dextran, affirming the formation of a functional epithelium within the breathing lung chip, with no significant difference observed between the two culture conditions (Figure 3D). Additional evaluations using ELISA assessed mucin MUC5AC secretion on the apical surface and the release of pro-inflammatory cytokines IL-8 into the medium layer. The secretion of MUC5AC gradually decreased by approximately 40% following ALI culture under breathing conditions compared to cells cultured statically (Figure 3E). In addition, the release of pro-inflammatory cytokines IL-8 decreased by 27% under breathing conditions (Figure 3F). These findings diverge from studies using one-way airflow under low shear stress (τ = 0.1 dyn/cm^2^), where mucin secretion increased with immediate exposure to airflow and showed no difference after a 24-h recovery period.^28^ The discrepancy in mucin secretion could be influenced by factors such as moisture or stress acting on the cells.^29^ Furthermore, the reduced IL-8 levels indicate that breathing conditions enhance cell viability and exhibit an anti-inflammatory tendency. Enhanced cellular responses associated with the breathing cycle may facilitate better nutrient and oxygen delivery, thereby mitigating inflammatory mediator release.^30^

### 3.4 Airway Inflammation Disease Modeling

**Figure 4:**
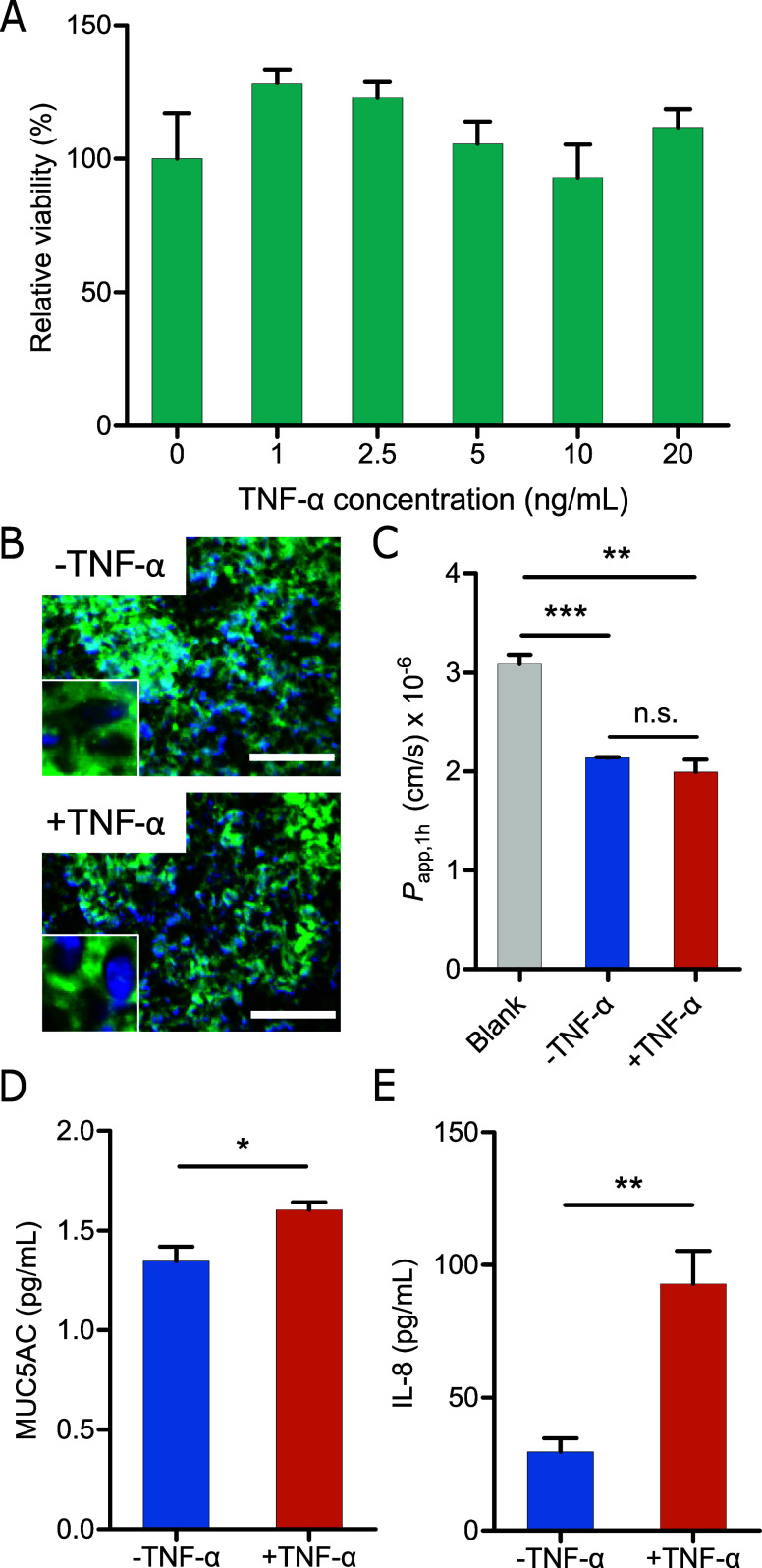
Diseased model in the breathing lung. (A) The relative viability of Calu-3 treated with TNF-α under different concentrations for 24 h. (B) Immunofluorescence staining images of ZO-1 (green) in DAPI (blue)-stained Calu-3 cells without and with TNF-α treatment. Scale bar = 100 μm. (C) Permeability of FITC-dextran for Calu-3 cells without and with TNF-α treatment. (D) MUC5AC secretion of Calu-3 cells without and with TNF-α treatment. (E) IL-8 is released from Calu-3 without and with TNF-α treatment. The concentration of TNF-α used in all experiments presented in (B–E) was 20 ng/mL. *n* = 3. *p < 0.05, **p < 0.001, ***p < 0.0001.

Typical airway inflammation responses manifest through various symptoms, including goblet cell hyperplasia, excessive mucus secretion, and elevated levels of pro-inflammatory cytokines such as IL-6 or IL-8. As these cytokines are primarily produced by lung parenchymal cells, our aim was to simulate this process by introducing a TNF-α containing medium into the medium layer on day 5 of ALI culture, maintained for a period of 1 day. Notably, TNF-α did not induce cell death among the Calu-3 cells; instead, it increased their relative viability within the breathing chip, particularly at low concentrations (Figure 4A). This effect can be attributed to TNF-α’s role in initiating signaling pathways that regulate cellular responses to stress and inflammation. At low concentrations, TNF-α can activate pro-survival signaling pathways, enabling cells to adapt to their environment and withstand mild stressors.^31^ Additionally, the observed increase in viability could reflect upregulated metabolic activity in response to the treatment, which is a common cellular reaction to stressors.

Immunofluorescence staining results in Figure 4B demonstrate that the cytoplasmic expression of the ZO-1 tight junction protein remained unaltered when TNF-α was introduced after 24 h. Similarly, the introduction of TNF-α did not affect the permeability of FITC-dextran, indicating that the Calu-3 cells retained their membrane integrity (Figure 4C).

To further assess the effects of TNF-α, an ELISA was employed to evaluate mucin MUC5AC secretion on the apical surface and the release of pro-inflammatory cytokine IL-8 into the medium layer following 24 h of TNF-α treatment. These assessments revealed a significant increase in MUC5AC secretion (*p* < 0.05) upon TNF-α stimulation (Figure 4D). Additionally, the release of pro-inflammatory cytokine IL-8 increased by a factor of 3 compared to the group without TNF-α treatment (Figure 4E). These outcomes underscore that introducing TNF-α in the medium layer of the breathing chip can effectively trigger inflammatory effects, thereby establishing an inflammation lung model suitable for drug screening.

### 3.5 Evaluation of Breathing Motion Effect

**Figure 5:**
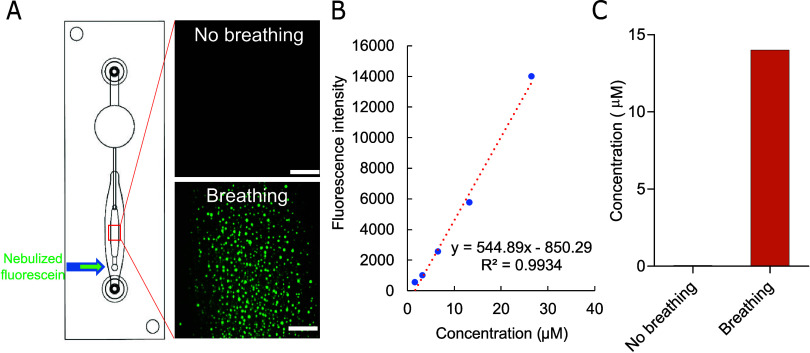
Nebulized fluorescein distribution in the breathing lung chip. (A) The fluorescence images of nebulized fluorescein distribution in the air layer where the cells were seeded with and without breathing motions. Scale bar = 200 μm. (B) The calibration curve of different concentration of nebulized fluorescein inhaled in the breathing lung chip. Each data point was analyzed *via* spectrometer (excitation = 485 nm; emission = 520 nm). (C) The calculated concentration of fluorescein deposited in the breathing lung chip with and without breathing motions after 10 min of breathing. All experiments were conducted using an airflow rate of 724 μL/min (or 2.23 cm/s) for breathing culture conditions.

To evaluate the effect of breathing motion and estimate the concentration of the drug inhaled into the breathing chip under physiologically relevant conditions, we implemented a verification process to quantify the drug dosage delivered by the nebulizer. Fluorescein was selected as it offered ease of quantification using a spectrometer and image analysis. Nebulization of fluorescein with a known concentration occurred in the inhalation exposure chamber (Figure S7A,B), followed by 10 min of breathing motion initiated by the pump. The condensation of nebulized fluorescein within the cell-seeded air layer of the chip was observable using a fluorescence microscope (Figure 5A). To quantify the collected condensed fluorescein, 100 μL of PBS buffer was introduced into the air layer, and the resulting mixture was measured using a spectrometer. This process enabled the creation of a calibration curve based on the known concentration of fluorescein collected from the breathing chip (Figure 5B), confirming that nebulized fluorescein could be inhaled into the lung chip through the breathing mechanism over 10 min (Figure 5C). As a result, the actual concentration of inhaled fluorescein within the lung chip could be calculated, with the total quantity determined as 527 ng for a concentration of 14 μM. In this experiment, we quantified the drug deposition and found a notable increase in delivery efficiency, with at least 200-fold difference observed between the breathing and nonbreathing conditions. This highlights the importance of respiratory dynamics in enhancing drug deposition within the lung model.

### 3.6 Comparison of Inhaled Drug Treatment

To ensure consistency in the quantity of nebulized drug deposited on both the breathing lung chip and the transwell, therefore, the total quantity of nebulized drug deposited on the transwell could be determined by establishing a calibration curve with various known concentrations (Figure S8A,B). The calibration curve was systematically assessed with varying exposure times of nebulized drug deposition on the transwell, providing corresponding drug concentrations (Figure S8C). For example, a 10 min nebulization period for the breathing lung chip equated to a 9-s direct exposure on the transwell.

**Figure 6:**
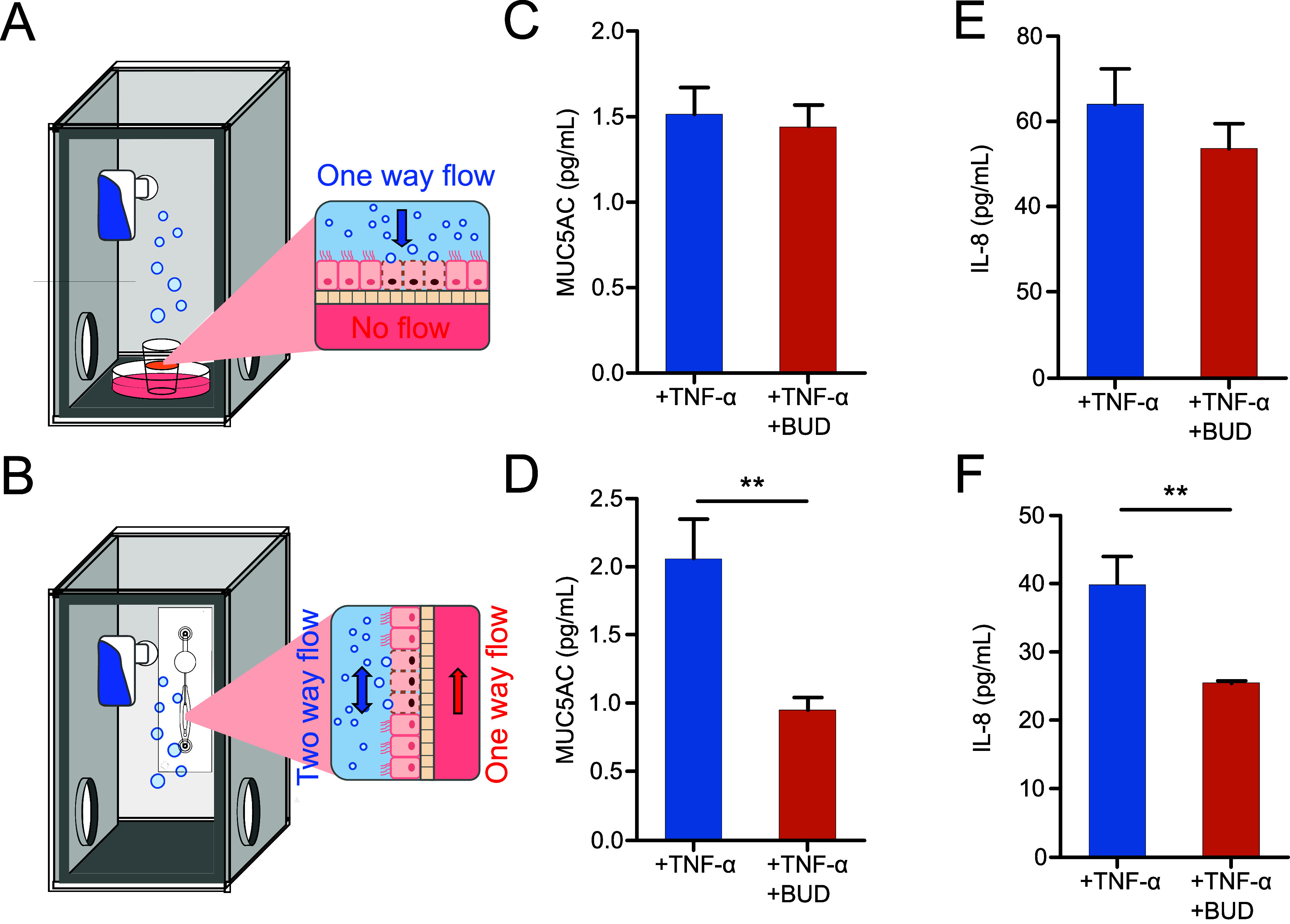
(A) Inhalation exposure chamber contains a transwell to study the direct exposure of the drug on the cells. (B) The inhalation exposure chamber contains a breathing lung chip to study the drug inhaled in the breathing lung chip. (C) MUC5AC secretion of Calu-3 cells without and with BUD treatment after TNF-α induction in the transwell. (D) MUC5AC secretion of Calu-3 cells without and with BUD treatment after TNF-α induction in the breathing lung chip. (E) IL-8 was released from Calu-3 without and with BUD treatment after TNF-α induction in the transwell. (F) IL-8 was released from Calu-3 without and with BUD treatment after TNF-α induction in the breathing lung chip. *n* = 3. *p < 0.05, **p < 0.001, ***p < 0.0001. Breathing lung chips were conducted using an airflow rate of 724 μL/min (or 2.23 cm/s).

After confirming the uniform distribution of nebulized fluorescein within the chip, we replaced the fluorescein solution with a drug solution, specifically budesonide (BUD), a corticosteroid drug used for the long-term management of COPD and other inflammatory disorders. The BUD solution was prepared in PBS with 0.1% DMSO to achieve a final concentration of 30 μM. We then examined the effects of nebulized BUD treatment on Calu-3 cells within the breathing lung chip. Immunofluorescence staining of the ZO-1 protein indicated the intact tight junctions after 24 h of breathing, followed by a 10 min BUD inhalation treatment (Figure S9). Comparison of MUC5AC secretion in TNF-α stimulated Calu-3 cells between direct exposure (Figure 6A) and inhalation with the breathing mechanism (Figure 6B) revealed a 7% decrease after direct nebulized BUD treatment (Figure 6C), while cyclic inhalation of nebulized BUD resulted in a significant 55% decrease in MUC5AC secretion (Figure 6D). These findings suggest that BUD treatment reduces MUC5AC secretion in TNF-α stimulated Calu-3 cells, with a more pronounced reduction when delivered with the breathing mechanism.

Moreover, a substantial decrease in IL-8 release of approximately 37% was observed in the breathing lung chip (Figure 6E) compared to cells a 17% decrease in cells subjected to direct exposure to BUD (Figure 6F), indicating the anti-inflammatory effects of BUD. Notably, a prior study demonstrated that treatment with 100 nM BUD using medium flow for 24 h could only reduce IL-8 release by 14% in another lung-on-a-chip model.^26^ Differences in efficacy between the two administration methods may arise from the back-and-forth airflow, enhancing drug delivery and cellular uptake.

While this study successfully establishes a diseased model for assessing drug efficacy, addressing drug toxicity comprehensively remains crucial. Future investigations should incorporate healthy lung models derived from stem cells, alongside primary human bronchial epithelial cells and microvascular endothelial cells, to provide a more accurate representation of lung physiology. This approach would enable a balanced assessment of both drug safety and efficacy, ensuring that potential adverse effects are thoroughly evaluated in both pathological and healthy contexts. Additionally, the low throughput of the platform, typical of many organ-on-a-chip systems, limits its scalability for high-throughput drug screening, which could pose challenges for industrial applications. While our current work focuses on small molecules, there is also potential to adapt the platform for the delivery of larger therapeutic agents, such as mRNA or antibodies, though further optimization of conditions will be required.

We primarily utilized immunofluorescence staining to evaluate the barrier integrity of the cultured Calu-3 cells. However, we recognize that additional methodologies could provide further insight. For instance, while we initially attempted to perform trans-epithelial electrical resistance (TEER) measurements, the current chip design does not support the use of larger electrodes required for accurate readings. As a result, we have relied on permeability assays to assess barrier function. Looking forward, the integration of custom electrodes directly onto the chip could allow for TEER measurements in future studies, offering a real-time assessment of barrier integrity.

Collectively, these results underscore the effectiveness of inhaled drug administration within our airway inflammation model and highlight the potential of this system as a promising platform for inhaled drug screening. Further improvements in chip design and throughput capacity will be essential for enhancing the platform’s utility in both preclinical and industrial drug development settings.

## 4 Conclusions

In this paper, the breathing lung chip model marks a pioneering advancement in respiratory research, seamlessly integrating airway-on-a-chip technology with dynamic breathing mechanisms. Rigorous experimentation with the Calu-3 cell line underscores the model’s viability, tight junction integrity, and relevance for studying respiratory diseases and drug responses. The introduction of TNF-α as a pro-inflammatory mediator validates its utility for airway inflammation studies. Notably, investigations into drug inhalation, particularly BUD, reveal the breathing mechanism’s pronounced enhancement of inhaled drug delivery efficacy. The calibration of drug dosage using fluorescein ensures precise drug delivery and accurate effect evaluation within the model. This breathing lung chip model emerges as a versatile platform for studying airway epithelium responses to inhaled drugs, inflammation, and disease mechanisms. Its potential impact on future drug development and therapeutic strategies targeting respiratory diseases is considerable. This research contributes advanced tools for respiratory studies and opens new avenues for understanding, treating, and managing respiratory diseases more effectively. Collectively, the breathing lung chip model stands as a promising and impactful innovation in respiratory research, offering a unique platform to bridge the gap between *in vitro* and *in vivo* studies, thus advancing our understanding and intervention strategies for complex respiratory diseases.

## Supporting Information Available

Design and image of breathing chip; the setup of airflow characterization; the cell seeding process; the setup of inhalation chamber; the calibration curve of the peristaltic pump; the fluorescence images of live and dead cells; the nebulized drug concentration analysis; the nebulized drug concentration analysis in the transwell; the additional immunofluorescence staining image of Calu-3 cells (PDF)
Measurement of airflow rate based on the moving distance of the oil plug in a silicone tubing. The tubing was connected to the airflow port for measurement purpose (Video S1) (MP4)
Breathing lung chip system connected with a circulating medium flow can be used for ALI cell culture (Video S2) (MP4)
Breathing motion of the breathing lung chip driven by inflation and deflation of the PDMS membrane (Video S3) (MP4)
