# Supplementary material for: A Dynamic Breathing Lung Chip for Precise Evaluation of Inhaled Drug Efficacy and Airway Epithelial Responses
Source: ACS Biomater Sci Eng. 2024 Dec 1;11(1):682–91. doi: 10.1021/acsbiomaterials.4c01377 (PMC11733924; doi:10.1021/acsbiomaterials.4c01377)
Supplement: Supplementary file 1 — ab4c01377_si_001.pdf [file ab4c01377_si_001.pdf]

Supplementary Information

A Dynamic Breathing Lung Chip for Precise Evaluation of Inhaled Drug Efficacy and Airway Epithelial Responses

Chao-Yu Liu[1], Ying-Ru Chen[1], Hsuan-Yu Mu[1], Jen-Huang Huang*[1]

[1]Department of Chemical Engineering, National Tsing Hua University, Hsinchu, 30013 Taiwan

Video S1. The measurement of airflow rate based on the moving distance of the oil plug in a silicone tubing. The tubing was connected to the airflow port for measurement purpose.

Video S2. The breathing lung chip system connected with a circulating medium flow can be used for ALI cell culture.

Video S3. The breathing motion of the breathing lung chip driven by inflation and deflation of the PDMS membrane.

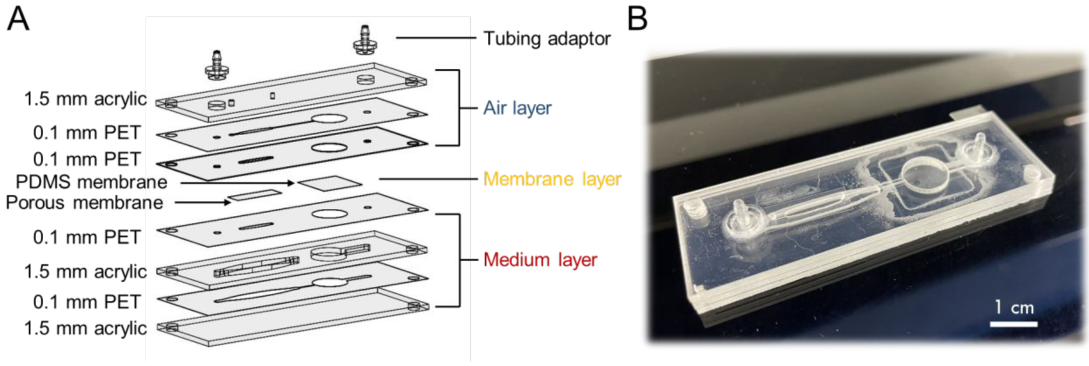

Figure S1. The breathing lung chip. (A) The design of breathing lung chip. The chip is composed of three layers: air layer, membrane layer, and medium layer. (B) The image of fabricated breathing lung chip.

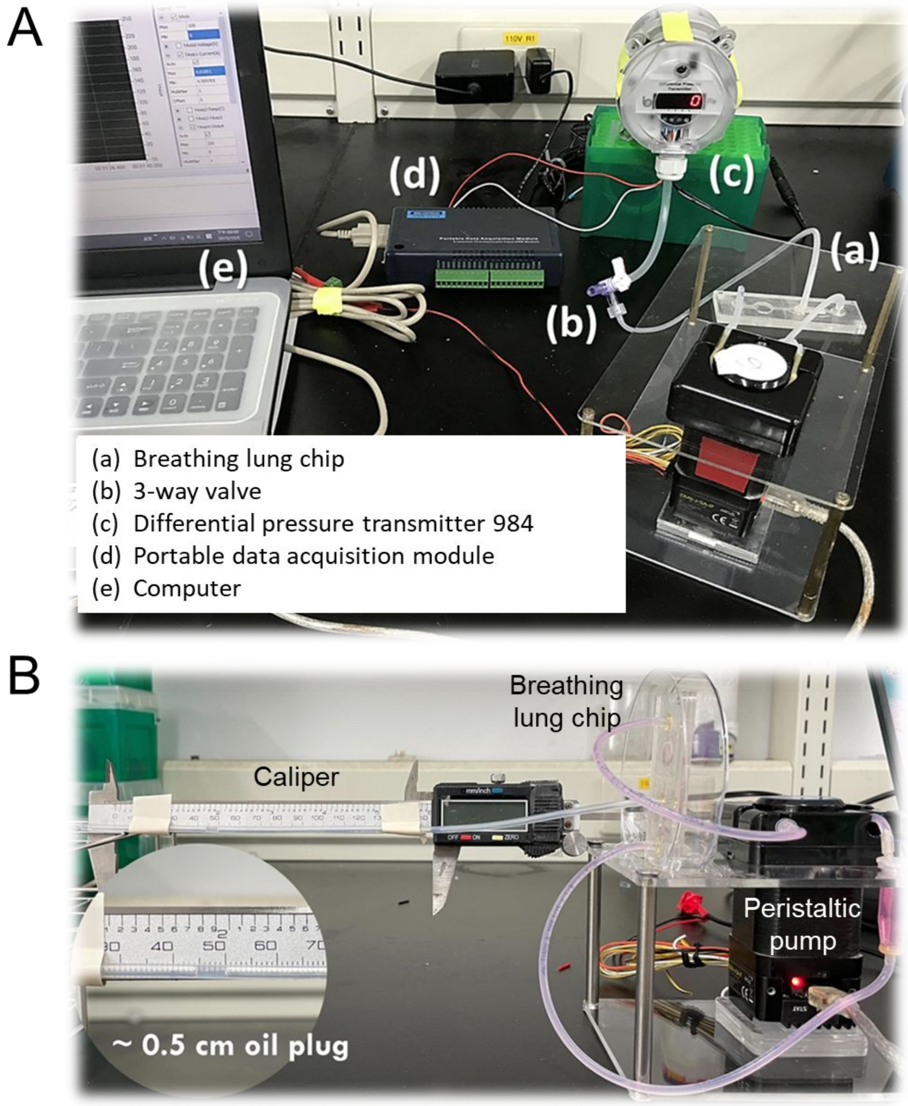

Figure S2. Setup of airflow characterization. (A) The setup of airflow pattern characterization using differential pressure sensor. (B) The setup of airflow measurement using oil plug movement analysis.

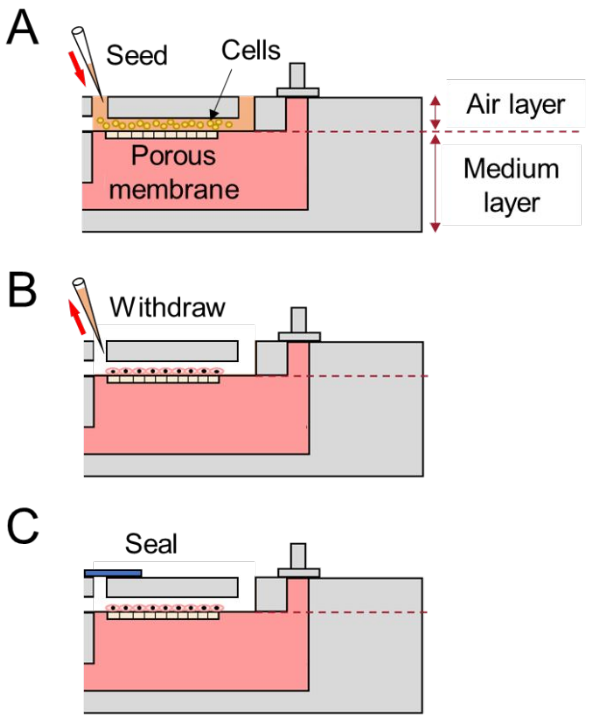

Figure S3. Cell seeding process. (A) The Calu-3 cells were seeded on the porous membrane of the chip through the seeding hole and cultured at liquid-liquid interface for 1 day. (B) After the cells were attached on the porous membrane, the medium was removed to allow the cells for air-liquid interface culture. (C) The cell loading hole was sealed before applying cyclic breathing motion.

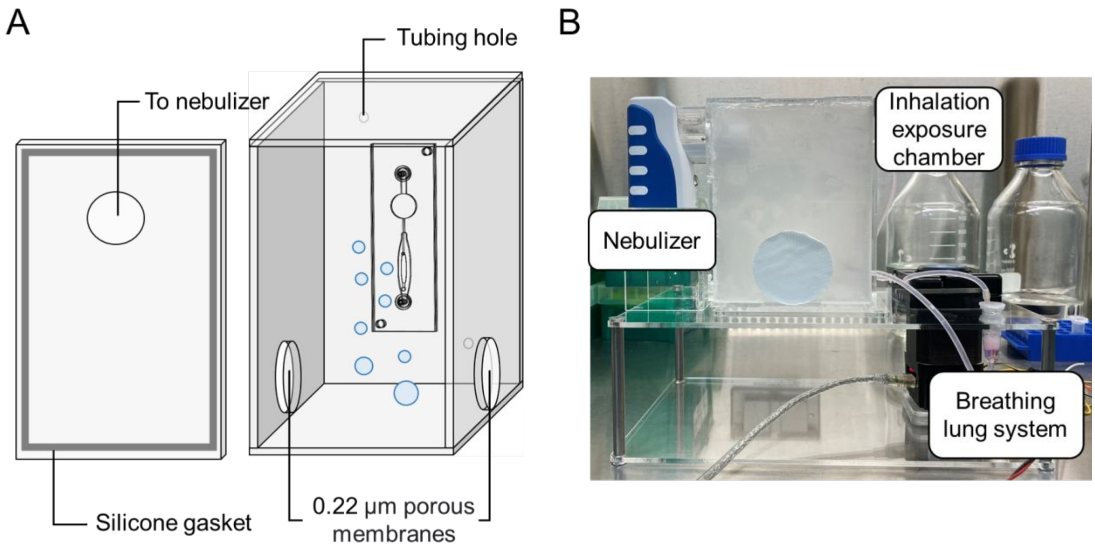

Figure S4. Setup of inhalation chamber. (A) The schematic of inhalation exposure chamber. The chamber has silicone gasket and porous filter to create an isolated and air pressure balance environment for inhalation drug test. The chamber was customized, allowing to connect a nebulizer and store a breathing lung chip to mimic the real treatment setup. (B) The image of inhalation exposure chamber, nebulizer and breathing lung system.

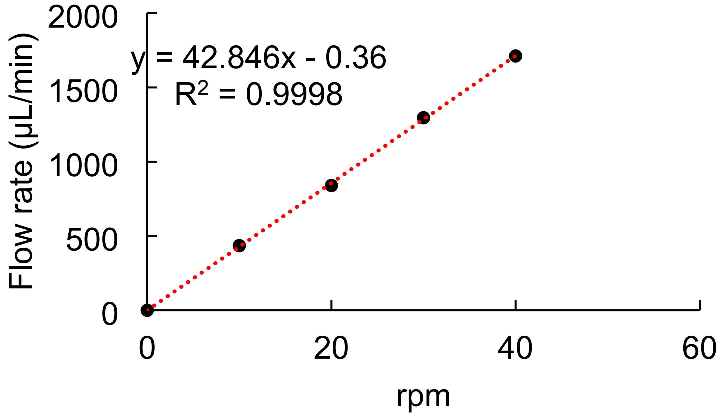

Figure S5. Calibration curve of the peristaltic pump. The flow rate was obtained by measuring the volume of fluid (i.e. water) at certain time.

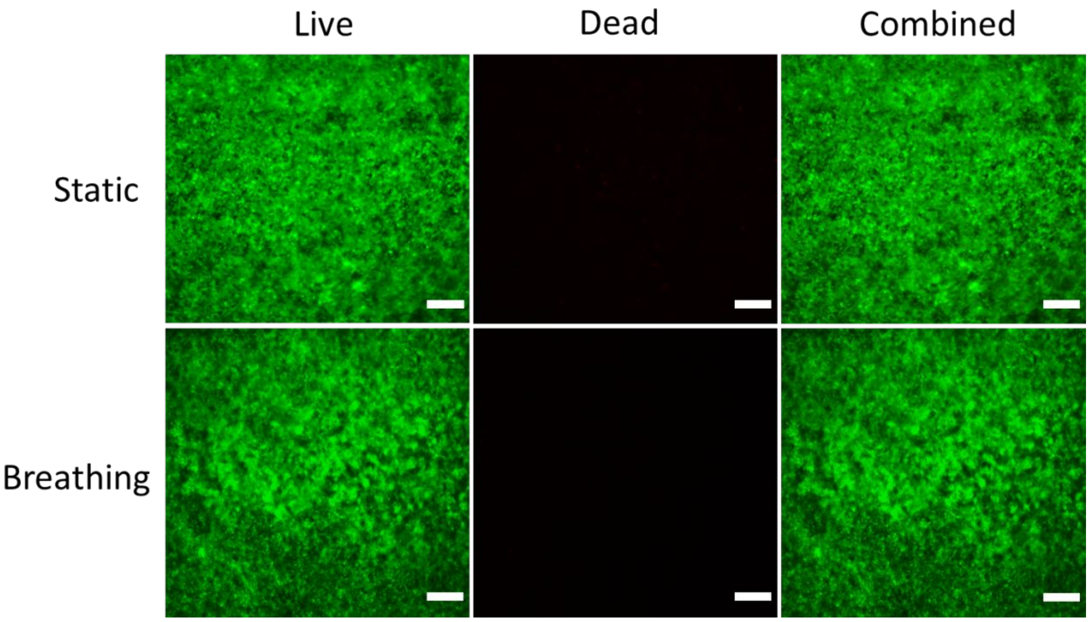

Figure S6. The fluorescence images of live and dead cells cultured at static condition (without breathing) and breathing culture condition for 4 days of ALI culture. Green: live cells; red: dead cells. Scale bar = 200 μm.

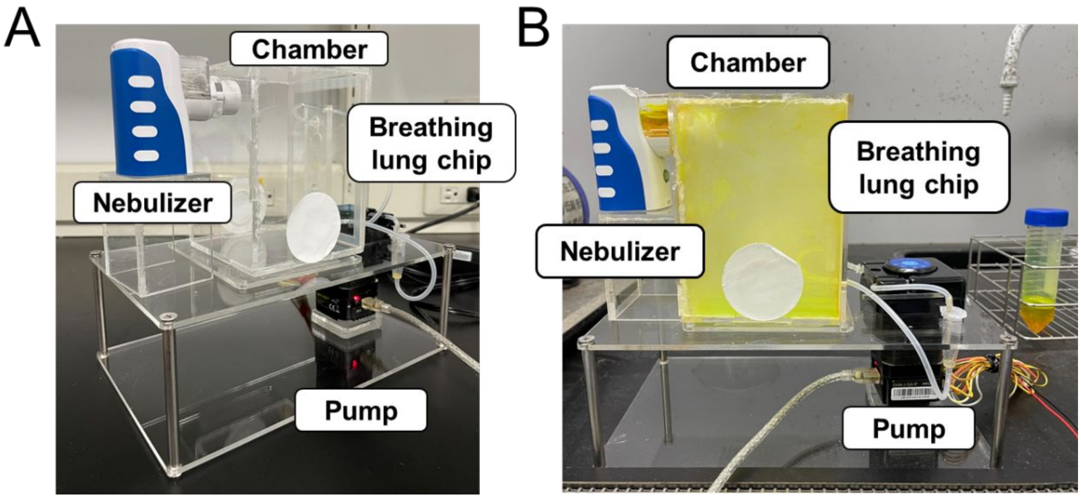

Figure S7. Nebulized drug concentration analysis in the breathing lung chip. (A) The setup of nebulized drug concentration analysis system before the nebulization of fluorescein. (B) The setup of nebulized drug concentration analysis system after the nebulization of fluorescein.

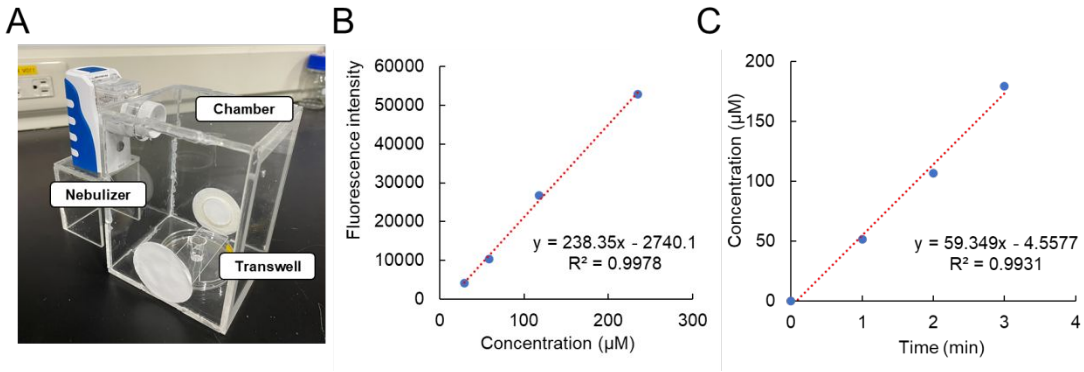

Figure S8. Nebulized drug concentration analysis in the transwell. (A) The image of nebulized drug concentration analysis using the transwell. (B) The calibration curve of different concentration of nebulized fluorescein deposited on the transwell. Each data point was analyzed via spectrometer (excitation = 485 nm; emission = 520 nm). (C) The calculated concentration of fluorescein deposited on the transwell after different time period.

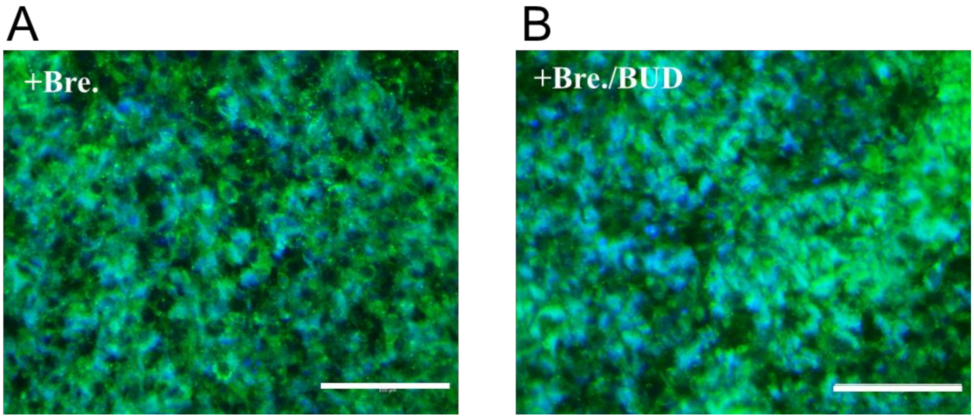

Figure S9. (A) The immunofluorescence staining image of ZO-1 (green) in DAPI-stained (blue) Calu-3 cells after 24 h of breathing. (B) The immunofluorescence staining image of Calu-3 cells after 24 of breathing followed by treating with nebulized BUD for 10 min. Scale bar = 100 μm.
